# Sex Hormones Regulate Cytoskeletal Proteins Involved in Brain Plasticity

**DOI:** 10.3389/fpsyt.2015.00165

**Published:** 2015-11-20

**Authors:** Valeria Hansberg-Pastor, Aliesha González-Arenas, Ana Gabriela Piña-Medina, Ignacio Camacho-Arroyo

**Affiliations:** ^1^Departamento de Biología, Facultad de Química, Universidad Nacional Autónoma de México, Mexico City, Mexico; ^2^Departamento de Medicina Genómica y Toxicología Ambiental, Instituto de Investigaciones Biomédicas, Universidad Nacional Autónoma de México, Mexico City, Mexico; ^3^Unidad de Investigación en Reproducción Humana, Instituto Nacional de Perinatología-Facultad de Química, Universidad Nacional Autónoma de México, Mexico City, Mexico

**Keywords:** sex hormones, estradiol, progesterone, brain, glial cells, plasticity

## Abstract

In the brain of female mammals, including humans, a number of physiological and behavioral changes occur as a result of sex hormone exposure. Estradiol and progesterone regulate several brain functions, including learning and memory. Sex hormones contribute to shape the central nervous system by modulating the formation and turnover of the interconnections between neurons as well as controlling the function of glial cells. The dynamics of neuron and glial cells morphology depends on the cytoskeleton and its associated proteins. Cytoskeletal proteins are necessary to form neuronal dendrites and dendritic spines, as well as to regulate the diverse functions in astrocytes. The expression pattern of proteins, such as actin, microtubule-associated protein 2, Tau, and glial fibrillary acidic protein, changes in a tissue-specific manner in the brain, particularly when variations in sex hormone levels occur during the estrous or menstrual cycles or pregnancy. Here, we review the changes in structure and organization of neurons and glial cells that require the participation of cytoskeletal proteins whose expression and activity are regulated by estradiol and progesterone.

## Introduction

Sex steroid hormones are known to play an important role during development and adulthood, regulating different functions and features of the central nervous system (CNS), such as brain differentiation, reproductive behavior, learning, and memory as well as neuroprotection. Structural plasticity is highly involved in the functional adaptation of the CNS in response to different environmental and physiological stimuli, including changes in hormone levels. In particular, female sex hormones can modify the size, morphology, synaptic density, and function of neuronal cells as well as the morphology of glial cells in sex steroid-responsive structures of the CNS (1). These changes are due to modifications in the neuronal and glial cytoskeleton where intracellular signals converge to regulate the direction and speed of outgrowth of different cell structures. Actin filaments, microtubules (MTs), and intermediate filaments, as well as the proteins associated with them, play a major role in synapse and dendritic spine formation (2). Neuronal projections are not only dependent on neuronal activity but also reliant on glial cells. The glia has an essential role in regulating the activity of CNS, where a mutual communication between glial cells and neurons exists. The activity and modifications in glial cell morphology also affect the formation and maintenance of synaptic contacts (3, 4). In this review, we will focus on the effects of female sex hormones on the expression and regulation of cytoskeletal proteins, contributing to the remodeling of the adult brain.

## Sex Hormones and the Brain

Female sex hormones are known to have a wide range of effects in the brain regulating not only reproductive processes but also cognitive functions. Estradiol (E2) and progesterone (P4) are cholesterol-derived hormones that, given their lipophilic structure, can easily cross the blood–brain barrier and interact with their specific receptors in different target cells of the brain. These hormones are also synthesized inside the brain. P4 and E2 levels have been detected in different brain areas such as hypothalamus and hippocampus with concentration differences between female and male animals (5–7), and their synthesis in neurons and glial cells have been demonstrated (8, 9). Moreover, pregnenolone, a cholesterol metabolite used by neurons for the biosynthesis of P4 and E2, is also produced by the glia (10, 11). This implies that the actions of sex hormones in neuronal plasticity are the result of adrenal, gonadal, and brain local synthesis.

E2 and P4 effects depend on the signaling pathway they activate, which can be either through intracellular receptors (classical mechanism) or membrane receptors (nonclassical mechanism) (12). Female sex hormone receptors are expressed in different brain areas, such as the hippocampus, hypothalamus, cortex, cerebellum, medial amygdala, substantia nigra, and ventral tegmental area (13–18). In the classical mechanism of action, sex hormones enter the cell and interact with their intracellular receptors, progesterone receptors A and B (PR-A and PR-B), and estrogen receptors α and β (ERα and ERβ). In the cell, the receptors are associated with chaperones like the heat shock proteins 70 and 90 (Hsp70/90). The ligand–receptor interaction induces conformational changes in the latter that promotes the receptor phosphorylation, dissociation of the Hsp70/90 complex, and dimerization. The active receptor binds to specific DNA sequences named hormone response elements (HREs) located within the regulatory regions of target genes. The receptor also recruits coactivators and chromatin remodeling complexes that enhance the attachment of the basal transcription machinery to induce gene expression. Genes that lack HRE can be hormonally induced through the interaction of the receptor with transcription factors like Sp1 and Ap1 (19–22). Once the receptor dissociates from the DNA, it is marked for degradation through the 26S proteasome (23, 24).

Hormone receptor activation can also induce diverse signaling pathways like those mediated by MAPKs, PI3K/Akt, and PKC (25–27), regulate second messenger cascades (28) or modulate the actions of neurotransmitter receptors (29). These mechanisms are regulated through PR and ER located in the cytoplasm, nucleus, or plasma membrane (30–32) or through other membrane receptors that have different biochemical and pharmacological properties (33, 34). These signaling pathways may eventually induce gene transcription. The different mechanisms of action of sex hormones may account for the diverse signaling profiles observed in various brain regions.

The effects of E2 and P4 in the brain depend on hormonal levels and receptor expression. The levels of P4 and E2 fluctuate throughout the life span of the rat modifying different parts of the CNS and causing diverse alterations in brain anatomy, physiology, and behavior (35, 36). E2- and P4-induced plasticity occurs when neuronal cells dynamically respond to hormonal stimuli by modifying its connectivity network and biochemical composition. Brain plasticity can be long lasting, and even the same stimuli can induce different plastic responses at different ages (37). The most dramatic change induced by sex hormones in brain is the driving of its sexual differentiation. During the fetal–neonatal period, sex hormones permanently modify the brain architecture (13, 38). Neurogenesis, cell differentiation, synaptogenesis, axon guidance, myelination, cell migration, and cell death are some of the main mechanisms occurring during sexual differentiation of the brain. These mechanisms alter the brain area, volume, cell number, cytoarchitecture, cell activity, synaptic connectivity, and neurochemical content (1, 39).

After brain differentiation, sex hormone levels in the brain are transitory and fluctuating, and induce the continuous functional adaptation of the CNS throughout the life span of the animal, particularly in females (35, 40). The main periods where sex hormone levels fluctuate during the life span are the beginning of puberty, reproductive cycles, pregnancy, and menopause. During these phases, alterations in the number of neurons and synapses, glial complexity, morphological variations in dendrites and synapses, and changes in neurotransmitter levels have been reported (41–44). These changes promote neuronal and glial remodeling that is critical for cognition, learning, and memory. For example, spatial working memory varies during rat pregnancy, and the memory retention enhanced by E2 is maintained by P4 (45, 46). Further data show that E2 and P4 modify neuronal morphology of the hippocampus of rats and monkeys, an important region for memory consolidation (47, 48). It has been recently reported that P4 enhances object recognition memory consolidation through mTOR and Wnt signaling (49). There is also evidence that both E2 and P4 can modulate GABAergic, dopaminergic, glutamatergic, and serotoninergic neurotransmission, as well as the release of a variety of growth factors from the astroglia (50–52). Sex hormones also modify the outgrowth of astrocytic processes and the amount of neuronal membranes they can cover, facilitating neuronal synaptic connectivity and plasticity (3, 51, 53). Morphological changes induced by E2 and P4 in the brain as well as the cytoskeletal proteins participating in brain plasticity, which are modulated by sex hormones, are reviewed in detail in the next sections.

## Sex Hormones Modify the Number, Size, and Biochemical Characteristics of Dendritic Spines

The effects of E2 and P4 on neuronal plasticity are related to adaptive changes in the structure and function of neurons that may contribute to learning, memory, and recovery after diverse insults (1). Reorganizational effects of sex hormones on neuronal circuitry involve different morphological events, including changes in dendritic length (54, 55) and neuronal membrane organization related to synaptic and dendritic spine formation (56). Dendritic spines, first described by Ramón y Cajal in 1888 (57, 58), are small protrusions of the dendritic membrane of neurons that are specialized in synaptic transmission. They consist of an actin-rich head attached to the neuron by a thin neck and contain the necessary postsynaptic machinery to receive the input of an excitatory synapse. Dendritic spines and synapses can be stable or change dynamically, even in very short time lapses, in their morphology and biochemical composition upon different stimuli (59, 60). Sex hormones have been shown to alter the structure and function of these neuronal structures through both rapid and long-term mechanisms (32, 61).

Recent studies show that E2 can modify synaptic plasticity and dendritic spine formation in hippocampal neurons through rapid signaling cascades, such as MAPKs, PI3K/Akt, and PKC (43, 62), which can also involve the activation of ERα (63–65). Signaling pathways such as ERK1/2 and Akt have been reported to be essential for E2-mediated spinogenesis in primary cortical neurons, and the activation of ERβ can mimic the rapid E2-induced spinogenesis and synaptogenesis. These results suggest that in cortical neurons, E2 via ERβ promotes neuronal cell remodeling by increasing the number of excitatory synapses (66). The same study showed that 30 min of E2 treatment induces the recruitment of postsynaptic density protein 95 (PSD-95) to the newly formed dendritic spines, while in the nascent, synapses promotes the recruitment of the *N*-methyl-d-aspartate (NMDA) receptor subunit GluN1 (66). These proteins are essential for the formation of new synaptic contacts, suggesting that E2 promotes the recruitment of the required proteins to allow pre and postsynapses to form connections. Other studies show that E2 promotes the phosphorylation of NMDA receptors through the activation of the src tyrosine kinase/MAPK pathway, and thus enhances long-term potentiation (LTP) of synapse transmission (67). Also, cyclic changes in E2 levels during the estrous cycle of rats are associated with changes in the state of NR2 subunit tyrosine phosphorylation of NMDA receptors in the hippocampus and alter LTP (68). In addition to E2, rapid effects of P4 have been reported in primary cultures of cortical neurons, where P4 increases the density and number of dendritic spines through changes in cell cytoskeleton components (69). The rapid effects of P4 on dendritic spines have been proposed to occur through the activation of GABA receptors and through the recently described PR membrane component 1 (65, 70).

Non-rapid effects of sex hormones in the brain have also been observed, and reports show that they induce the formation of excitatory synapses both *in vitro* and *in vivo* (47, 48), thus modulating LTP (71, 72). For example, ovariectomized rats treated with E2 for 48 h showed an enhanced density of apical dendritic spines in the CA1 region of the hippocampus that was related to an increase in the number of functional synapses (73). Interestingly, the density of dendritic spines in the hippocampal pyramidal cells changes during the estrous cycle of the rat; more spines are observed during the afternoon of the proestrus and the morning of the diestrus when E2 and P4 levels are high (5, 74). Moreover, Kato and colleagues demonstrated that the concentration of E2 in the hippocampus correlates with the serum concentration observed during the estrous cycle (5). However, hormone levels in the brain vary between newborn female and male animals (7, 75), suggesting the importance of considering the developmental stage and sex of the animal for a better evaluation of the observed hormone effects. Other studies show that adult male rats have more spines than female animals in the medial nucleus of the amygdala, and that the density of these spines varies throughout the estrous cycle of virgin rats, showing fewer spines during the proestrus and estrus phases when compared to diestrus (76, 77). Remarkably, the inhibition of E2 synthesis in females but not in males results in LTP and synapse loss in hippocampal slices (78, 79), which points toward an important effect of local E2 synthesis on synaptic plasticity.

E2 also induces the formation of neural pathways during fetal and neonatal life that modulate the activity of synapses in adulthood (80). The role of P4 in synaptic plasticity is less studied, but it has been reported that in cerebellar slices of neonatal rats, P4 promotes dendritic outgrowth and synaptogenesis in Purkinje neurons contributing to the formation of new neuronal connections in this structure (81). Immature cerebellar Purkinje cells treated with P4 for 24 h increased the dendritic length and spine density but this effect was not observed in mature cells. The effect was blocked when cells were treated with PR antagonist RU486, which suggest a classical mechanism of action for this hormone in the cerebellum (70). Interestingly, chronic treatment with P4 (60 days) decreases hippocampal synaptic transmission and LTP in hippocampal slices from ovariectomized adult rats (65). These data suggest that in mature cells, P4 effects on dendritic spine formation and LTP are less clear than for E2. With respect to the importance of the glia, primary cultures of rat astrocytes treated with P4 for 24 h express higher levels of agrin, a protein shown to be important for synapse formation. The P4-induced increase in agrin in astrocytes enhances synapse formation in hippocampal neurons (82). These data show the strong relation between glia and neurons that can be modulated by sex hormones. Many of these changes observed in the adult brain eventually converge on the cell cytoskeleton. Neuronal and glial cytoskeletal reorganization depends on its own dynamic nature and on the expression, regulation, and activity of the proteins associated with it.

## The Cytoskeleton in Neuronal Plasticity

The neuronal cytoskeleton is divided into three specific structural complexes with different properties: neurofilaments (NFs) or intermediate filaments, MTs, and microfilaments (MFs), each one with a specific composition and organization, and even a particular cell type or subcellular localization. NFs are heteropolymers composed of heavy, medium, and light NFs protein chains. NFs are very abundant in neuronal axons and have extremely elastic fibrous properties that help to maintain the asymmetrical shape of the neuronal cell and to regulate the axon diameter and growth (83). In addition to NFs, MTs are mainly located in the neuronal axon, where microtubule-associated proteins (MAPs) like Tau help to stabilize them. MTs are composed of heterodimers of α and β tubulin that give them an intrinsic polarity important for their dynamic nature (84). MTs and their MAPs (MAP1B, MAP2, etc.) participate in the promotion of neurite extension, the induction of distinctive morphologies between axons and dendrites, axonal transport, neuronal plasticity, and neuronal degeneration (85). Lastly, MFs are constituted by actin filaments, and their polymerization dynamic is closely associated with the activity of actin-binding proteins (ABPs) like drebrin and ADF/cofilin. MFs are involved in a broad range of aspects that are crucial for the establishment and the correct function of synapses, axonal cones growth, shape, size, remodeling of dendritic spines, and protein trafficking (86).

The neuronal cell shape, the dendritic spines, and synapse morphology, as well as the speed of synapse growth, can be hormonally modulated (87–89). Morphological changes depend on the cell cytoskeleton, and its dynamic regulation helps to shape these diverse neuronal structures. Experimental evidence suggests that MFs and MTs play a prominent role in the establishment and stability balance in neuronal structures, such as synapses and dendritic spines, which are constantly constructed and modified throughout life (90, 91). Dendrites and their spines have important implications in neuronal activity. Cytoskeleton studies show that MFs are highly accumulated in the dendrite spines where a pool of dynamic MFs is located at the tip of the spine, while a pool of stable drebrin–actin filaments is located in the spine core. These stable drebrin–actin filaments interact with dynamic MTs whose presence is enhanced by synaptic activity. The interplay between MFs and MTs is therefore important for the temporal and local regulation of spine morphology (2, 92, 93). These cytoskeleton rearrangements are controlled by members of the Rho family of GTPases (e.g., RhoA, Rac1), which regulate the activity of different cytoskeleton-associated proteins such as MAPs and ABPs (94, 95).

**Figure 1:**
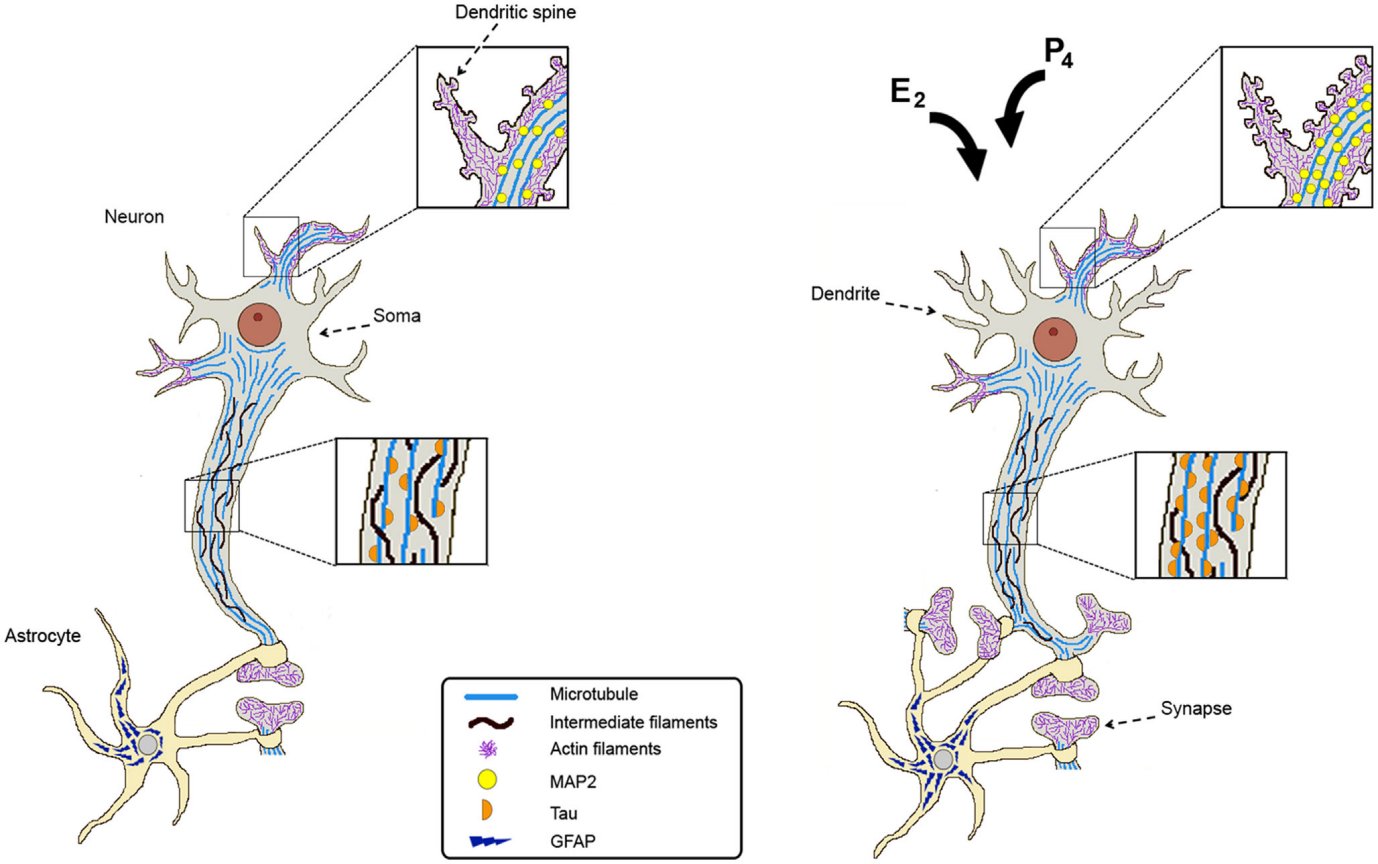
**Estradiol and progesterone regulate the expression of cytoskeletal proteins and promote neuronal plasticity**. Cytoskeletal proteins are indicated as follows: microtubules at dendritic spines, soma, and axon; intermediate filaments along the axon; actin filaments at dendrites; Tau along the axon, MAP2 at dendrites and dendritic spines; and GFAP in astrocytes. After estradiol (E2) and/or progesterone (P4) treatments, cytoskeletal proteins increase their content in a region-specific manner and this correlates with an increase in the number of dendrites, dendritic spines, and synaptic contacts. Hormonal stimuli also increase astrocytes ensheathing the synapses providing the support for the organization and well functioning of synaptic connections.

Synaptic connections are very important for neuronal communication, so they are highly regulated. Astrocytes are active players in neuronal transmission and plasticity. They can extend their projections to surround neuronal somata, dendrites, and synapses. Actually, the majority of synapses are ensheathed by astrocytes providing the support for the organization and well functioning of the synaptic connections (Figure 1) (96). Astrocytic processes have in their structure bundles of intermediate filaments constituted by glial fibrillary acidic protein (GFAP). These projections gradually form a network that infiltrates the brain tissue in order to effectively associate with neuronal synapses (97, 98).

Different cytoskeletal proteins are modified when morphological plastic changes occur in the brain in response to diverse stimuli. Gonadal sex hormones are known to affect diverse morphological processes as mentioned in the text, so we will further review the effects of E2 and P4 on three of the main cytoskeletal proteins present in CNS cells.

## E2 and P4 Promote the Remodeling of the Actin Cytoskeleton

Actin is a highly conserved protein involved in many important cellular processes, including contraction, cytokinesis, transport of vesicles and organelles, cell signaling processes, establishment and maintenance of cell junctions and cell shape, cell movement, and synaptic plasticity (99, 100). These actin features are mainly attributed to filamentous actin, which represents the major cytoskeletal component of dendritic spines (101). Hence, the morphological changes in spine shape, size, and number are determined by local actin dynamics (102). The overall process of cytoskeleton remodeling, including the formation of new MFs and their interaction with the plasma membrane, depends on the participation of diverse ABPs.

P4 and E2 are key modulators of cell morphology and movement in diverse cellular types, including neurons (103–106). Most of the events leading to cytoskeletal rearrangement are rapidly performed by changes in the phosphorylation state of ABPs. A key protein that controls actin remodeling is the WASP-family verprolin homologous protein 1 (WAVE1) whose activation by phosphorylation is essential to regulate actin polymerization through the actin-related protein Arp2/3 complex (107, 108). In this regard, E2 and P4 administration to rat cortical neurons leads to WAVE1 phosphorylation on 310, 397, and 441 serine residues. Phospho-WAVE1 is then redistributed toward the cell membrane at the sites of dendritic spine formation. An ERα rapid extranuclear signaling activates GTPase Rac1, which recruits the cyclin-dependent kinase 5 triggering WAVE1 phosphorylation. E2 also induces actin remodeling via the activation of ABP moesin through the RhoA and ROCK2 pathway (109). Moesin phosphorylation on Thr558 is essential to link the actin cytoskeleton to a variety of membrane-anchoring proteins, such as CD43 and CD44 (110, 111). Rat cortical neurons treated with E2 and P4 exhibit an increase in phosphorylation of moesin, which impacts the formation of neuronal spines (69, 109). Actin polymerization in dendritic spines of rat hippocampal slices has been linked to E2 activation of RhoA pathway that leads to the inhibition of the filament-severing protein cofilin (112). Interestingly, treatment of hippocampal slices with aromatase inhibitor letrozole promotes actin filaments depolymerization as a result of cofilin activation, thus leading to synapse loss (113). Also, it has been reported a transient spine density increase in cortical neurons treated with E2 dependent on a Rap/AF-6/ERK1/2 pathway (114). Another study reported that E2 induced an increase in the length of dendrites in the central nucleus of the amygdala and in the hypothalamic ventromedial nucleus that was due to the inactivation of cofilin and variations in the composition of GluA1 and GluA2 subunits of the AMPA receptors (87). The changes in the actin cytoskeleton suggest a possible relation between dendrite and dendritic spine remodeling and changes in animal behavior regulated by E2.

There is evidence that demonstrates that P4 increases the outspread of the neuronal growth cones of dorsal ganglia neurons, an effect related to morphological changes in the components of the actin cytoskeleton. The enhanced cytoskeletal dynamic within the growth cone after P4 treatment occurred through a classical mechanism of action because the effect was blocked by the administration of PR antagonist RU486 (115). These data show that E2 and P4 induce morphological changes in shape, size, and number of neuronal spines, and that these changes are determined by actin dynamics, suggesting a continuous plastic transformation (Figure 1).

## E2 and P4 Effects on Microtubule-Associated Protein 2 and Tau Expression

Microtubule-associated proteins regulate MTs dynamics by selectively binding to distinct conformations of polymerized and unpolymerized tubulin. Among them, the structural MAPs stabilize the MTs by binding along the length of the MT (116). In the brain, the main structural MAPs are MAP1, MAP2, and Tau, each one presenting several isoforms. Neuronal MAPs are differentially expressed during brain development: MAP1B is expressed in early stages of newly forming axons, MAP1A is expressed in mature axons, and both MAP2 and Tau isoforms are expressed during development and adulthood, predominantly in dendrites and axons, respectively (117, 118). In particular, Tau isoforms are of clinical relevance, given that they are the major component of paired helical filaments found in Alzheimer’s disease (AD) and other brain diseases (85, 119).

It has been reported that MAP2 is preferentially located at the shaft of dendrites, where it may have the capacity to regulate morphological plasticity at a slow rate when compared to the rapid morphological changes regulated by actin filaments in dendritic spines (91). In the CA1 region of the hippocampus of MAP2-deficient mice, apical dendrites were shorter than those of wild-type animals (120, 121), suggesting an important role for MAP2 in dendrite elongation.

The expression pattern of MAPs and their correlation with ultrastructural changes induced by ovarian steroids have been observed in different brain areas and under specific hormonal and developmental conditions (89, 122–124). In medial basal hypothalamic neurons maintained for 4 days *in vitro* (DIV), E2 increased the levels of the 58-kDa Tau isoform but it did not change that of tubulin; by 7 DIV, E2 also increased the content of MAP1 and MAP2 (125). In cultured hypothalamic dissociated neurons, E2 exerted differential effects on neurite outgrowth depending on gender: the induction and differentiation of axons occur later in time, and cells develop fewer and shorter primary neurites in female fetuses compared with neurons taken from male fetuses. A comparable increase in Tau and MAP2 expression was observed in neuronal cultures obtained from both female and male rats (126). Another study showed that in dissociated cell cultures form embryonic rat medial amygdala, E2 induces the differentiation of axons after 21 DIV and increases the total dendritic length of the cultured neurons. These changes were correlated with the respective increase in Tau and MAP2 expression but not with that of α-tubulin (127).

In the hippocampus of ovariectomized rats, an increase in MAP2 protein content has been reported after the treatment with E2, P4, or both hormones for 24 and 48 h, with no changes in the frontal cortex. Interestingly, these hormones did not modify MAP2 mRNA content in the hippocampus. These data suggest that MAP2 is involved in the structural changes induced by E2 and P4 in hippocampus and that its expression is regulated at a postranscriptional level (123). Interestingly, it has been demonstrated that the chronic administration of ovarian hormones immediately after ovariectomy modifies the content of MAP2 and Tau in the hippocampus and prefrontal cortex of the rat. Short- (2 weeks) and long-term (18 weeks) treatments with E2 or P4 had different and even opposing effects on MAP2 and Tau expression. None of the proteins changed its content in the prefrontal cortex in response to E2, but remarkably, P4 decreased MAP2 after short-term treatment and increased both MAP2 and Tau in this brain region after a long-term treatment. In the hippocampus, short- and long-term treatments with E2 increased MAP2 content, while P4 did it only after a short-term treatment (128). These data suggest that P4 regulates MAP2 expression depending on the brain region and the exposure time to the hormone, and it would be interesting to study P4 effects in E2-primed animals. Other authors have found similar tissue-specific effects with P4. For instance, in ovariectomized rats, an acute injection of P4 had no effect on Tau expression in the hypothalamus after 24 h, while it induced a decrease in the cerebellum (129). Another study reported that after P4 treatment for 3 days, the loss of MAP2 induced by acute spinal cord injury was attenuated, suggesting that P4 is partially responsible for preserving neuronal ultrastructure at the peripheral level (130). These studies highlight the importance of the type and length of treatment, the doses of E2 and P4 used and as well as the brain region studied; a summary of these results are shown in Table 1.

**Table 1:** **Changes in MAP2A and Tau protein content in the hippocampus and frontal cortex of ovariectomized rats after acute and chronic E2 and P4 treatments**.

Brain area	Time of treatment	E2		Time of treatment	P4		Reference
		MAP2A	Tau		MAP2A	Tau	
Hippocampus	48 h	Increase	Increase	24 h	Increase	Increase	Reyna-Neyra et al. (123)
Frontal cortex		NC	NC		NC	NC	
Hippocampus	2 weeks	Increase	NC	2 weeks	Increase	NC	Camacho-Arroyo et al. (128)
Frontal cortex		NC	NC		Decrease	NC	
Hippocampus	18 weeks	Increase	NC	18 weeks	NC	NC	Camacho-Arroyo et al. (128)
Frontal cortex		NC	NC		Increase	Increase	

*The study of two brain regions and the modifications in protein content after acute (24 and 48 h) and chronic (2 and 18 weeks) hormone treatments*.

*NC, no change in protein content*.

During pregnancy, circulating sex hormones are increased in the rat; E2 levels are two-fold and P4 three-fold higher compared with the hormone levels during proestrus day (131, 132). The brain displays diverse morphophysiological changes during pregnancy including cell plasticity (36, 45, 133). Furthermore, in the medial preoptic area (POA), late pregnant rats have bigger neuronal somata than ovariectomized rats (134), suggesting that E2 and P4 play an important role in neuronal morphology. Changes in the expression of MAP2 and Tau in the hippocampus and POA were evaluated during rat gestation and the beginning of lactation. In the hippocampus of pregnant rats, the content of MAP2 decreased during pregnancy, contrary to ovariectomized rats treated with P4 during 2 weeks (128, 132). These differences in P4 effects suggest a very fine regulation of MAP2 protein expression that depends on the characteristics of the hormonal stimulus. In addition, no significant changes in MAP2 content were detected in POA through rat pregnancy, suggesting that tissue-specific factors are involved in the regulation of MAP2 expression (132), which could be related to the different roles that have specific brain areas in the behavioral patterns observed throughout pregnancy.

Differences in Tau protein content and in its phosphorylation pattern in different brain regions may be related to Tau key role in the dynamic remodeling of neuronal cytoskeleton observed during gestation. Tau content and its phosphorylation are modified in a tissue-specific manner in the pregnant rat (132). In the hypothalamus, the hippocampus, and the cerebellum, Tau content diminished on gestation day 14 compared to gestation day 2, and only in the cerebellum and the hippocampus, this decrease was sustained until day 18 of pregnancy. Phosphorylated Tau at Ser396 (PhosphoTau) progressively augmented in the hippocampus, the hypothalamus, and the cerebellum throughout pregnancy, whereas in POA, the content of PhosphoTau decreased on day 21 of gestation (135). Tau phosphorylation at Ser396 results in tubulin depolymerization and MTs destabilization (136). Recent data show that Tau has an important role in synaptic plasticity in the hippocampus and that Ser396 phosphorylation is required for long-term depression (LTD), which is associated with the weakening of synaptic connections (137). LTD is important for certain cognitive processes like novelty discrimination and behavioral flexibility (138), which are fundamental for the pregnant rat.

Changes in MAP2 and Tau expression have been seen even after days of E2 or P4 treatment (1 day and 18 weeks), suggesting a classical mechanism of action where intracellular PR and ER are involved. However, not only MAP2 and Tau are under sex hormones influence, there are other proteins involved in synaptogenesis (neuroligins) or in spine density formation (PSD-95), whose expression also depends on P4 and E2 levels. Neuroligin-2 expression in the uterus is upregulated after 3 days of treatment with E2, P4, or E2 + P4 (139). Six-hour of E2 treatment stimulates the phosphorylation of Akt, as well as the phosphorylation of the translation initiation factor 4E binding protein 1. In turn, the activation of these signaling intermediates promotes the increase in the translation of PSD-95 in cultured neuronal cells (140). These data demonstrate that E2 and P4 induce the expression of different proteins involved in neuronal plasticity by different mechanisms of action.

## Sex Hormones and Their Impact on Glial Fibrillary Acidic Protein Expression

Nowadays, it is evident that astrocytes respond to various stimuli by increasing their intracellular calcium levels, releasing gliotransmitters (141) or rapidly extending their projections (97). The large astrocytic processes have bundles of intermediate filaments that have GFAP as one of their principal constituent. GFAP has been implicated in cell motility (142), astrocyte proliferation (143), directional mobility of vesicles (144), the integrity of the blood–brain barrier, myelination (145), neuroprotection, and brain plasticity (146, 147).

Glial fibrillary acidic protein expression can be modified by factors such as neuronal damage, stress, age, or hormones (148). Sex hormones can regulate the astrocyte number during rat hippocampal development (149), enhance the extension of GFAP immunoreactive processes in astrocytes from hippocampal slices *in vitro* (150), and modulate astrocyte reaction after brain injury (151, 152). Interestingly, GFAP fluctuates during the estrous cycle of the rat and has a marked sex difference, at least in the hippocampus. The CA1, CA3, and dentate gyrus regions of the hippocampus had an increase in GFAP immunoreactivity during proestrus (high levels of P4 and E2) compared to male animals and diestrus females. During proestrus, astrocyte morphology changed to rounded cell bodies with numerous and short processes, whereas cells with stellate shape with few and long processes were present in the hippocampus of males and diestrus females (153).

During pregnancy and the beginning of lactation, a differential expression pattern of GFAP was found in the brain. Gómora-Arrati and coworkers analyzed GFAP expression on days 2, 14, 18, and 21 of gestation and the second day of lactation (L2) of the rat because of the marked changes in E2 and P4 levels observed in these days. It was found that in the hippocampus, GFAP content showed a constant increase of 25% throughout pregnancy and L2, while in the cerebellum, it first decreased more than 30% during pregnancy and later increased on L2 (41%). Interestingly, GFAP content increased in the frontal cortex and hypothalamus on gestational days 14 and 18, respectively. Then, a subsequent decrease was observed in the following days of pregnancy that persisted until L2 in the hypothalamus, in the cortex increased (42%) in L2. Contrary, a dramatic decrease in GFAP content was observed in POA on day 14 followed by an increase that was maintained throughout the rest of the studied days. These data suggest a differential expression of GFAP that should be associated with changes in brain function during these reproductive stages (154). Other reports showed that the chronic administration of P4 in ovariectomized rats resulted in a reduction of GFAP content in the hippocampus (128). This result contrasts with that observed under physiological conditions, highlighting the importance of hormonal concentration and exposure time on the content of GFAP in the brain.

E2 also modulates astrocytic form and function in the hypothalamus of rodents during development and adulthood. In the developing arcuate nucleus, E2 increased stellation of astrocytes through increases in neuronal GABA synthesis (155). Likewise, E2 positively regulates the length of GFAP-positive processes through ERα activation in astrocytes of ovariectomized animals (156). Still, there is no evidence whether E2-induced changes in astrocytes morphology are indirect effects of the E2 stimulation of neighboring neurons. Other reports show that in ovariectomized rats with entorhinal cortex lesions, E2 replacement inhibits the increase in GFAP (mRNA and protein level) and enhances neurite outgrowth. It is proposed that the decrease in GFAP alters the organization of laminin and this increases the fibrillary extracellular matrix supporting axonal growth (157). In adult castrated male rats, GFAP expression increased in the hippocampus, however, high levels of E2 prevented this castration-induced increase in GFAP (148). Interestingly, as evidence described herein shows that most of the effects of steroid hormones on GFAP expression are long term, and the data suggest that both P4 and E2 dynamically modify both the content and the distribution of GFAP.

## Sex Hormones in Neurogenesis, Neuroprotection and Disease

E2 and P4 have been shown to exert both neuroprotective and neuroregenerative roles in several models of brain damage (158–161). Neurogenesis in the adult animal occurs in the cells lining the subventricular zone and the dentate gyrus of the hippocampus, where cells can remain quiescent or be activated to finally produce neuronal progenitor cells that later migrate into diverse brain regions (37, 162). It has been observed that neurogenesis in the dentate gyrus is higher in female animals than in males, probably because of the variations in gonadal hormones (163). Also, chronic treatment (21 days) of ovariectomized rats with E2 + P4 increased neurogenesis in the dentate gyrus (164). Regarding brain damage, E2 can induce neurogenesis post stroke in the adult animal (165) that could be through the activation of ERs (166, 167), and P4 has been reported to increase neurological functions after a traumatic brain injury (168). As a prerequisite for neuronal transmission, the new neurons need to have a well-defined axon and dendrites, which is known as neuronal polarization. The cytoskeleton is fundamental for the process of neuronal polarization (169) and as described in this review, sex hormones can modulate the expression and regulation of important proteins of the cytoskeleton.

The neuroprotective effects of sex hormones have been observed under different brain insults and diseases. In an ischemic model, P4 reduces neurite growth inhibitory proteins like RhoA and Nogo-A, and E2 diminishes the loss of neurons and synapses from de CA1 hippocampal region (170, 171). In neurodegenerative diseases like AD, E2 administration reduces the expression of β-amyloid precursor protein, which is cleaved into amyloid beta (Aβ) and accumulated in plaques in the brain (172, 173). Aβ is involved in the generation of AD and it has been reported that estrogens can reduce its concentrations in the brain (174). Also, the formation of tangles of Tau protein caused by its abnormal phosphorylation, another AD characteristic, has been shown to be counteracted by E2 (175, 176). In fact, some studies have demonstrated that E2 therapy reduces the risk of presenting this neurodegenerative disease in women as well as diminishes the cognitive impairment associated with it (177, 178). Neuroprotective properties of E2 and P4 have also been observed *in vitro* in neuronal models of cell death induced by glutamate in hippocampal and cortical neurons (179, 180). Both E2 and P4 can induce recovery from neurodegeneration by increasing the synthesis of myelin components in both Schwann cells and oligodendrocytes (10, 181, 182). In fact, P4 promotes the expression of the myelin basic protein in cultured rat oligodendrocytes (183, 184). Taken together, sex hormones promote the recovery of brain tissue upon an insult and also protect against neurodegenerative diseases.

## Conclusion

E2 and P4 play a key role in different neuronal and glial cell functions that involve changes in synaptic plasticity, and therefore in cell structure (Figure 1). These sex steroids induce changes in the brain cells cytoskeleton in addition to the content and activity of cytoskeletal proteins, such as MAP2, TAU, and GFAP. However, these changes significantly vary depending on sex, age, cerebral region, as well as the dose and length of exposure to these hormones.

## Perspectives

There are several promising research areas that will give us a better understanding of the participation of sex steroid hormone action in cytoskeletal proteins regulation. The knowledge of the action mechanisms used by sex hormones to modulate cytoskeleton and therefore synaptic plasticity will be important to understand how learning and memory skills change during puberty, reproductive cycle, pregnancy, lactation, and menopause.

## Conflict of Interest Statement

The authors declare that the manuscript was prepared in the absence of any commercial or financial relationships that could be construed as potential conflicts of interest.
